# Diseases of the Fingers

**Published:** 1891-09-12

**Authors:** 


### SURGERY.

### DISEASES OF THE FINGERS (*continued*).

#### F.—Abnormalities of Circulation.

(1) Chilblains.—People with languid and feeble circulation and those whose occupations subject them to sudden alternations of temperature are the most apt to have chilblains. It is not correct to consider them indicative of struma. The condition is one of stasis in the blood vessels over a circumscribed area. The early stages of inflammation, viz., dilatation of the arterioles and slowing of the blood current occur and there is effusion of serum, but instead of this leading to pus formation or active effusion and distension of the tissues, there seems to be a local paralysis of vessels and an almost complete stoppage of the circulation. Whether this is due to thrombosis or to pressure from the poured out serum is not clear. In order to prevent this affection the most important thing is to avoid the sudden warming of the fingers when they are cold. After handling ice-cold substances, especially, the return of heat should be retarded, not hastened. By this means the inflammatory reaction which is the essence of the process will be prevented. Amongst the glove-wearing portion of the community the constriction of the circulation by tight inelastic gloves is a common predisposing cause of chilblains. The best treatment is the application of warmth over the whole hand for a long period (not for only a minute or two) and the stimulation of iodine or friction. The Linimentum Tocli of the B. P. with a little extra iodine added, so as to make the strength one to seven or one to six is one of the best remedies. When the chilblains are broken warmth is still more needed, and a stimulating ointment such as the Unguentum Hydrarg: Ammon: with a little Balsam of Peru added.

(2) Raynaud's Disease.—This affection has many other names, such as "local asphyxia," "symmetrical gangrene," &c. It varies in its severity from mere "deadness" or lividity of the finger-tips to extensive gangrene of the hands. The observer, from whom it has received its name, describes the condition as produced by "some error as to the innervation of the capillary vessels." If this is the pathology, most people who have bathed in the sea in cold weather have suffered from it more or less; for it is common to have, under these circumstances, constriction of the arterioles in the extremities, which appear white or blue, and do not for a time regain their colour even after warming before a fire. Dyspepsia favours this temporary spasm, as does any intestinal irritation. If this condition continues, however, it becomes worthy of the name "Raynaud's Disease." It is highly probable, however, that many other causes besides contraction of the musculature of the small arteries will bring about the same result. Thus, "obliterative endarteritis," atheroma, and embolism may explain some cases of the disease. As pointed out by Dr. Jacoby (see paper read before New York Neurological Society, January 6th, 1891), the affection is not uncommon in syphilis, where the internal coat of the arteries is sometimes enormously thickened. Neuritis (peripheral) is probably the origin of the disease sometimes, and the condition known as "arterio-capillary fibrosis" may give rise to the same condition of "local asphyxia." Bright's disease is certainly a predisposing cause, no doubt chiefly through the arterial thickening associated with it. We must, therefore, consider the affection as sometimes local (when due to atheroma, embolism, or endarteritis) and sometimes general (as in Bright's disease, arterio-capillary fibrosis, and as a symptom of nerve disturbance). Dr. Jacoby points out that the local signs are identical in all these. The worst cases, however, must usually be those in which there is actual arterial obstruction, and, consequently, "dry" gangrene. When the disease is nervous, it may be due to three distinct conditions, viz , (1) peripheral neuritis, (2) central disease in the brain or spinal cord, or (3) reflex disturbance (as intestinal or stomachic). The treatment is only surgical in so far as the application of warmth to the part, with massage and counter-irritation, is concerned; and if gangrene ensues, in the removal of the necrosed tissues and the encouragement of healthy granulations.

(3) Morvan's Disease.—This peculiar condition consists in atrophy of the nails and ends of the fingers, with scarring and thickening of the skin and occasionally necrosis of the phalanges. It has been found (Bernhardt) in a case of disease of the vertebræ and in diseased conditions of the spinal cord and its membranes. In the *Archives de Medecine Experiment : et d' Anatomie Pathol :* 1890, a post-mortem examination on a subject of this affection is recorded. The meninges of the cord were thickened, and there was a cavity in the posterior columns, most marked in the cervical region. Dr. Joffry and Dr. Achard, who report the case, and Dr. Bernhardt consider the disease to be always due to central nerve lesions, and especially to syringomyelia. This being the case, the local treatment is unimportant, the part to be attacked being, obviously, the spinal cord and its coverings and bony framework, and perhaps in some cases the brain.

Although the above three conditions have been classified under the heading "Abnormalities of Circulation," it must be understood that this is merely for the sake of convenience, the pathology differing widely in each, and the lesions of the fingers being in many cases secondary to more important affections.

---

The manufacturer of olive oil seems to be almost as conservative in his ways as the potter who still uses the wheel of many centuries back. The mill in use to-day on the Mediterranean coast for the purpose of crushing the olives differs little from those used for centuries. An inventor of a new mill has now come forward, but as the people of the olive-raising countries are slow to adopt new ideas, it will probably be some time before the new invention supersedes the old-fashioned, but present, method.

